# Effects of swimming training on cecum microorganisms and metabolites in rats with high fat diet

**DOI:** 10.3389/fmolb.2025.1569239

**Published:** 2025-08-08

**Authors:** Chuan Dong, Ling Chen, Dengpan Li, Xuefeng Zhang, Yulong Chen

**Affiliations:** ^1^ Department of Physical Education, Gansu Agricultural University, Lanzhou, China; ^2^ College of Animal Science and Technology, Gansu Agricultural University, Lanzhou, China; ^3^ College of Food Science and Engineering, Gansu Agricultural University, Lanzhou, China; ^4^ College of Mechanical and Electrical Engineering, Gansu Agricultural University, Lanzhou, China

**Keywords:** high fat diet, swimming, SPF rat, metagenomics, metabonomics

## Abstract

Swimming is a whole-body aerobic exercise that has preventive and therapeutic effects on chronic metabolic diseases triggered by a high-fat diet. SPF grade rats (n = 48) were selected. They were divided into 4 groups (GB, GY, ZY and ZB) with 12 rats in each group. The GB and GY groups were fed high-fat chow during the pre-test period, and the ZY and ZB groups were fed normal chow. Swimming training was carried out in ZY and GY groups and no swimming exercise in GB and ZB groups in the later part of the trial. Histopathological staining was performed on the cecum and liver of 48 rats. Physiological and biochemical indices such as ACP, ALP and AST were measured in the blood of the rats in each group, and 6 samples of cecum contents were taken from each group for metagenomics and widely targeted metabolomics. The results showed that AST, ALP, ACP, LDL, IL-6, TNF-α, and IL-1β were significantly lower in the GY group than in the GB group; the structural liver lesions were severe in the GB and GY groups; and the ZY group had higher levels of *Prevotellaceae*, *Muribaculaceae*, and *Spirochaetes*. In comparison with the GB group, the GY group showed significant increases in metabolites associated with metabolic pathways such as ABC transporters and sulfur metabolism. The results show that feeding high-fat diet can cause tissue and organ lesions, cecal microbe and metabolite structure changes in rats. However, swimming training increased the content of beneficial microorganisms and metabolites in rats’ cecum. This study provides a theoretical basis for swimming exercise to alleviate metabolic disorders caused by high fat diet.

## 1 Introduction

In modern society, obesity has become a public health problem worldwide due to the accelerated pace of life and changes in eating habits. Obesity affects an individual’s appearance and mental health, and it is also closely related to a variety of chronic diseases such as heart disease, diabetes, and hypertension (Booth et al., 2019). One of the main factors contributing to obesity is a high-fat diet, which leads to excess calorie intake, which in turn causes weight gain and obesity (Li et al., 2020). In addition, a high-fat diet can further exacerbate metabolic disorders by altering the structure and function of the intestinal microbial community, triggering intestinal flora dysbiosis (Chen et al., 2024).

The intestinal microbial community, often referred to as the gut flora, is the group of microorganisms in the human gut, including bacteria, fungi, viruses, and parasites (Bai et al., 2022). The intestinal flora is the most numerically diverse microbial community in the human body, and they form a symbiotic relationship with the host and are deeply involved in regulating gene expression, gut barrier function, nutrition, metabolism, and overall host immune function. These microbes interact with the host and work together to maintain the health and stability of the gut (Paone and Cani, 2020). However, a high-fat diet can disrupt this balance, leading to an increase in harmful bacteria and a decrease in beneficial bacteria, thus triggering intestinal flora dysbiosis (Tong et al., 2021). When the balance of gut flora is disrupted, dysbiosis occurs, which may have negative effects on human health. Dysbiosis of the gut flora may lead to metabolic disorders, increasing the risk of metabolic diseases such as obesity and diabetes. Because gut flora are involved in the regulation of the host’s energy balance, and they promote fat synthesis and storage by fermenting food components which are not digested and broken down, and converting them to short-chain fatty acids. At the same time, a high-fat diet can affect Pan’s cell function, which in turn reduces the level of defensins and may lead to the disturbance of intestinal flora (Beam et al., 2021). A high-fat diet can also negatively affect the diversity and abundance of gut microbiota, while leading to elevated lipid levels in the blood (Wang et al., 2020). A high-fat diet also affects the metabolic activity of the intestinal flora, leading to a decrease in certain beneficial flora and an increase in harmful flora (Sun et al., 2023). To summarize, dysbiosis of intestinal flora may lead to metabolic disorders through various mechanisms, so it is very important to maintain the balance of intestinal flora for the maintenance of human health.

Swimming exercise is a full-body aerobic exercise that can help burn fat, lose weight and improve physical functioning (Rothberg and Makarewich, 2019). Several studies have shown that moderate swimming exercise can increase the diversity of intestinal microorganisms as well as the number and metabolic activity of beneficial bacteria, thereby positively affecting health (Lagier et al., 2015; Ramos, 2022). Moderate swimming exercise can increase the content of bifidobacteria in the intestinal tract of rats, while decreasing the content of intestinal bifidobacteria in diabetic rats (Duranti et al., 2020). Swimming exercise not only improves blood lipid levels in rats, but also positively affects the structure of intestinal flora (Basson et al., 2021). Swimming exercise can significantly increase the proportion of microorganisms responsible for the secretion of short-chain fatty acids in the intestinal flora of rats, which is involved in the regulation of endocrine secretion and the maintenance of energy homeostasis of the organism (Mountzouris et al., 2009). In addition, swimming exercise increased the abundance of Trichoderma spp. and Ruminalococcus spp. and promoted the increase of metabolite butyrate content, which regulated the intestinal pH value and reduced the risk of colorectal cancer (Godha et al., 2018). Swimming significantly improved the lipid metabolism disorder in rats, and this improvement may be related to its regulatory effect on the structure of intestinal flora (Olaniyi, et al., 2023). Swimming exercise can increase the diversity of intestinal flora and improve metabolic and immune responses, thus positively affecting lipid metabolism in rats. Swimming improves and regulates the activity of the immune system in rats and activates the production of molecules and metabolites of anticancer cells, thus inhibiting the growth of tumors (Greenfield, 2020).

In conclusion, swimming exercise may be beneficial in alleviating high-fat diet-induced gut flora dysbiosis and metabolic disorders in rats. In this experiment, we investigated the specific mechanism of swimming exercise on gut flora by macrogenomics and metabolomics approaches, and how to optimise the structure and function of gut flora by adjusting dietary and exercise habits to improve metabolic health.

## 2 Materials and methods

### 2.1 Ethics approval

All animals involved in the experimental procedures were approved by the Animal Care Committee of Gansu Agricultural University (GSAU-Eth-PE-2023-001), in compliance with the animal care and experimental procedure guidelines established by the Ministry of Science and Technology of the People’s Republic of China (Approval No. 2006-398).

### 2.2 Animals materials and experimental design

An 8-week-old male SD rats (n = 48, body weight 200 ± 10 g, SPF grade) were selected and purchased from the Laboratory Animal Center ofc Lanzhou Veterinary Research Institute, Chinese Academy of Agricultural Sciences [License No.: SCXK (Gan) 2020-0002]. These rats were randomly divided into 4 groups of 12 rats each after 7 days of acclimatization at the Animal Training Center of Gansu Agricultural University, which were high-fat-fed group with no swimming training (GB), high-fat-fed group with swimming training (GY), normal-fed group with swimming training (ZY), and normal-fed group with no swimming training (ZB). These rats were fed in separate cages with 4 rats per cage and 3 cages per group. The room temperature was set at 23°C ± 3°C, relative humidity 50% ± 5%, and 12-h day/night alternation. During the feeding period, rats in the ZY and ZB groups were fed according to the basal diet, and rats in the GY and GB groups were fed according to the high-fat diet, and all rats were fed and watered freely. The nutrient composition of the basal and high-fat diets is shown in Tables 1, 2.

**TABLE 1:** Nutritional composition of basic diet.

Item	Content	Item	Content
water	98 g/kg	Methionine + cystine	9.98 g/kg
crude protein	207.1 g/kg	Ca	15 g/kg
crude ash	71 g/kg	Ca:P	1.49:1
crude fiber	33 g/kg	lysine	13.44 g/kg
crude fat	43 g/kg	total phosphorus	10.1 g/kg

**TABLE 2:** Diet composition and nutrient composition of high fat diet.

Ingredient	Unit calorific value (kcal/g)	Weight (gram)	Quantity of heat (kcal)
Caisein	4	200	800
L-Cystine	4	3	12
Sucrose	4	68.8	275
Dyetrose	4	125	500
Lard	9	245	2,205
Soybean Oil	9	25	225
Cellulose	0	50	0
Mineral Mix#210088	1.6	10	16
Calcium Phosphate	0	5.5	0
Potassium Citrate H_2_O	0	13	0
Vitamin Mix # 300050	3.9	10	39
Choline Bitartrate	0	2	0
Blue Dye	0	0.05	0
total		773.85	4,072

Item	gm%	Kcal%
Protein	26	20
Carbohydrate	26	20
Fat	35	60
Kcal/gm	5.26	

The rats in the GY and ZY groups were subjected to non-weight-bearing swimming exercise every afternoon, and the exercise intervention was formally started after 3 days of acclimatization swimming practice. On the first day, the rats swam for 15 min, and then increased by 15 min every day for 1 week to 90 min, and then maintained this amount of exercise until the end of the experiment for 6 consecutive days per week. The swimming pool used for the test had a smooth inner wall, dimensions of 120 cm × 80 cm × 70 cm, and a water temperature of 31°C ± 1°C. The rats were observed for locomotion at all times during the test, and the non-swimming rat was driven away to prevent it from being in a stationary floating state.

### 2.3 Sample collection and processing

After 6 weeks of swimming training, all rats were executed by anesthesia. All rats were given 1–2 mL of blood. A small piece of tissue was cut from the middle of the liver and cecum and placed in a 15 mL centrifuge tube filled with 4% paraformaldehyde. The contents of the cecum were taken and loaded into 5 mL freezing tubes. After snap-freezing in liquid nitrogen, they were sent to Metavir Biotechnology Co. Ltd. (Wuhan, China) for Metagenomics and Metabolomics sequencing.

### 2.4 H&E stain

The liver and cecum specimens fixed with 4% paraformaldehyde were coated with paraffin wax, and paraffin sections with thickness of 5 µm were prepared. After HE staining, the morphology and structure of each section were observed under a 4× light microscope (Olympus BX43, Japan).

### 2.5 Measurement of physiological and biochemical indexes

The blood of 48 rats was collected, and the supernatant was transferred to the frozen storage tube after centrifugation. AST (U/L), ALT (U/L), ALP (U/L), ACP (U/L), TC (mmol/L), TG (mmol/L), HDL (mmol/L), LDL (mmol/L), IgM (g/L), IL-1β (pg/mL) and IL-6 were performed (pg/mL) and TNF-α (pg/mL) were determined.

### 2.6 Metagenomics sequencing

All samples (n = 24) were subjected to DNA extraction. DNA purity and integrity were analyzed by agarose gel electrophoresis (AGE), and DNA concentration was precisely quantified by Qubit 4.0 Fluorometer (Thermo Fisher Scientific, St. Louis, MO, United States). The qualified DNA samples were subjected to library construction and library testing, and the qualified libraries were sequenced by Illumina PE150, and the Raw Data obtained from sequencing was processed by removing splices and low-quality sequences to obtain Clean Data. After that, *de novo* assembly was performed (assembly parameters: -k-list 21,41,61,81,91 -min-contig-len 500), and the unutilized reads of each sample were put together for mixed assembly, in order to discover the low abundance species information in the samples (Maarala et al., 2017). The assembled contigs were subjected to CDS prediction and short fragments of contig sequences were filtered out (Gurevich et al., 2013). Sequence clustering was performed to obtain a set of non-redundant Unigenes, and the abundance of Unigenes in each sample was obtained. The Unigenes were compared with the MicroNR library to obtain species classification information at each level (Li et al., 2014). Alpha and Beta diversity analyses were performed on the annotation statistics of species at the gate level, and LEfSe (Linear discriminant analysis Effect Size) and metabolic pathway comparative analyses were performed for each group to explore the differences in species composition and functional composition among samples (Segata et al., 2011). Functional composition differences were analyzed by comparing Unigenes with functional databases such as KEGG (Kanehisa et al., 2006), eggnog (Huerta-Cepas et al., 2016), CAZy (Cantarel et al., 2009), CARD (Jia et al., 2017), GO (Brown et al., 2022), and so on, to obtain the annotation information of each database.

### 2.7 Metabolomics profiling

All samples (n = 24) were thawed on ice, weighed 20 mg (±1 mg) into the corresponding numbered centrifuge tubes; 400 μL of methanol-acetonitrile-water (3:3:4, v/v/v) internal standard extract [containing 20 μM deuterated butyric acid (D_9_), 10 μM deuterated leucine (D_3_), 5 μM deuterated adenosine (D_5_), 5 μM deuterated cholic acid (D_5_), and 2 μM deuterated palmitic acid (D_31_)] was added and vortexed for 3 min. If the sample was not dispersed, steel balls were added and vortexed for another 3 min; the samples were sonicated for 10 min in an ice-water bath; the samples were removed and vortexed for another 1 min, and then allowed to stand in the refrigerator for 30 min at −20°C. 300 μL of the supernatant was transferred into another pre-labeled centrifuge tube corresponding to the sample number; Centrifuge the sample at 12,000 rpm/min for 10 min at 4°C, then carefully transfer 300 μL of the supernatant into another pre-labeled tube; centrifuge the sample again at 12,000 rpm/min for 3 min at 4°C and transfer 200 μL of the supernatant into a tube lined with the corresponding injection bottle for on-line analysis. The data acquisition instrumentation system primarily consists of Ultra Performance Liquid Chromatography (UPLC) (ExionLC AD, https://sciex.com.cn/) and Tandem mass spectrometry (MS/MS) (QTRAP®, https://sciex.com.cn/). For UPLC, the chromatographic column was Waters ACQUITY UPLC HSS T3 C18 1.8 µm, 2.1 mm*100 mm; the mobile phase consisted of phase A ultrapure water with 0.1% formic acid and phase B acetonitrile with 0.1% formic acid. The elution gradient was set as 95:5 V/V water/acetonitrile at 0 min, 80:20 V/V at 2.0 min, 40:60 V/V at 5.0 min, 1:99 V/V at 6.0 min and 7.5 min, 95:5 V/V at 7.6 min and 10.0 min, with a flow rate of 0.4 mL/min, a column temperature of 40°C, and an injection volume of 2 μL. For MS/MS, the electrospray ionization source temperature was 500°C, the mass spectrometry voltage was 5500 V in positive mode and −4500 V in negative mode, ion source gas I was 55 psi, gas II was 60 psi, curtain gas was 25 psi, and the collision-activated dissociation parameter was set to high. In the triple quadrupole (Qtrap), each ion pair was scanned and detected based on optimized declustering potential and collision energy.

### 2.8 Data statistics and analysis

The experimental data were preliminarily organized by Excel 2016, and then analyzed by one-way ANOVA using the ANOVA process of SPSS 26.0 statistical software, and Duncan’s method for multiple comparisons test. The results were expressed as the mean ± standard deviation. *p* < 0.05 indicated significant differences. In addition, in the metabolomics data analysis, based on the self-constructed targeted specimen database MWDB (metware database), the qualitative analysis was performed based on the retention time RT (Retention time) of the detected substances, the daughter ion pair information and the secondary spectrum data. The Empirical Cumulative Distribution Function (ECDF) was used to analyze the frequency of occurrence of the CV (coefficient of variation) of substances smaller than the reference value (0.3). Principal component analysis was performed on each sample, and QC samples were monitored according to the PCA model. The relative content differences of metabolites in the two groups of samples and their significance were displayed using a Volcano Plot. KEGG pathway enrichment analysis was performed based on the differential metabolites. In addition, the Spearman correlation test was used to explore the correlation between cecum microorganism and metabolite content. The greater the correlation coefficient, the stronger the correlation between the variables.

## 3 Results

### 3.1 Histopathological changes of cecum and liver in rats after high fat diet or swimming exercise

**FIGURE 1:**
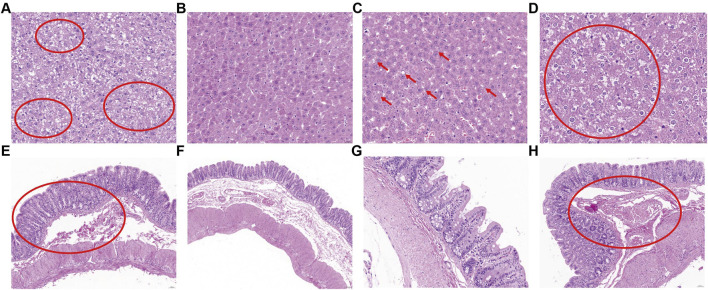
Histopathological section. **(A)** HE staining sections of liver tissue in GB group. **(B)** HE staining sections of liver tissue in ZY group. **(C)** HE staining sections of liver tissue in ZB group. **(D)** HE staining sections of liver tissue in GY group. **(E)** HE stained section of cecum tissue from GB group. **(F)** HE stained section of cecum tissue from ZY group. **(G)** HE stained section of cecum tissue from ZB group. **(H)** HE stained section of cecum tissue from GY group.

As shown in Figure 1. The liver lobules in the ZY group were clearly demarcated, all hepatocytes were polygonal, with clear cell borders, clear nuclei, homogeneous cytoplasmic staining, clear hepatic sinusoids, and no pathological damage such as steatosis was seen. In the GB group, the livers showed severe steatosis, the lobular demarcation was not clear, the hepatocytes were very swollen and the cells were filled with large amounts of free fat droplets of different sizes. The cells were filled with a large number of free fat droplets of various sizes, the nuclei of the cells were covered or squeezed to one side, the hepatic sinusoids were atrophied or even disappeared under pressure, and the hepatocytes with severe steatosis showed necrotic disintegration of the cells. The livers of the GY group showed more severe vesicular degeneration, and some of the hepatocytes were also accompanied by steatosis, and most of the hepatocytes underwent severe vesicular degeneration, and some of them were accompanied by steatosis. The cecum sections were then observed and analysed. The mucosal epithelium of the cecum in all groups was complete, highly columnar, with a smooth surface and no intestinal villi. There were a large number of cup-shaped cells interspersed among the mucosal epithelial cells, and there were no degenerated and detached epithelial cells in the intestinal lumen. There were no abnormal changes in the structure and cellular characteristics of the submucosa, muscularis propria and plasma membrane of the cecum in any of the groups. However, there was some oedema and bruising in the lamina propria of the cecum in the GB and GY groups, and no oedema and bruising in the lamina propria of the cecum in the ZB and ZY groups.

### 3.2 Changes of physiological and biochemical indexes in blood of rats after high fat diet or swimming exercise

As shown in Table 3, among liver function indexes, AST, ALT, ALP and ACP in GB group were significantly higher than those in ZY group and ZB group, while AST, ALP and ACP in GY group were significantly lower than those in GB group. In blood lipid indexes, TC, TG and LDL in GB and GY groups were significantly higher than those in ZY and ZB groups, and HDL in GB group was significantly lower than that in other groups. In terms of immune indexes, IgM levels in GB and GY groups were significantly lower than those in ZY and ZB groups. In terms of inflammatory factors, TNF-α, IL-1β and IL-6 in GB group were significantly higher than those in other groups, and TNF-α and IL-6 in ZY and ZB group were significantly lower than those in ZY and ZB group.

**TABLE 3:** Changes of blood physiology and biochemistry in rats.

Index	GB	GY	ZB	ZY	*P*-value
AST (U/L)	53.28 ± 2.69^a^	50.91 ± 0.44^b^	49.51 ± 1.09^b^	46.86 ± 2.89^c^	0.01
ALT (U/L)	28.40 ± 0.28^a^	28.39 ± 0.68^a^	25.39 ± 1.12^b^	21.96 ± 0.67^c^	0.01
ALP (U/L)	62.33 ± 1.47^a^	54.97 ± 1.14^b^	51.96 ± 2.13^c^	50.57 ± 1.16^c^	0.01
ACP (U/L)	9.96 ± 0.58^a^	8.44 ± 0.23^b^	8.04 ± 0.22^c^	7.79 ± 0.49^c^	0.01
TC (mmol/L)	0.50 ± 0.02^a^	0.51 ± 0.04^a^	0.44 ± 0.03^b^	0.39 ± 0.03^c^	<0.01
TG (mmol/L)	0.33 ± 0.02^a^	0.33 ± 0.01^a^	0.24 ± 0.03^b^	0.21 ± 0.01^c^	0.02
HDL (mmol/L)	0.24 ± 0.02^c^	0.30 ± 0.05^b^	0.32 ± 0.03^ab^	0.35 ± 0.02^a^	<0.01
LDL (mmol/L)	0.20 ± 0.01^a^	0.19 ± 0.01^b^	0.18 ± 0.01^c^	0.18 ± 0.01^c^	0.01
IgM (g/L)	0.34 ± 0.01^b^	0.34 ± 0.01^b^	0.49 ± 0.01^a^	0.49 ± 0.01^a^	0.01
IL-1β (pg/mL)	23.86 ± 0.52^a^	20.19 ± 1.04^b^	20.20 ± 0.52^b^	20.05 ± 0.49^b^	0.01
IL-6 (pg/mL)	119.41 ± 1.72^a^	113.18 ± 2.84^b^	105.27 ± 2.88^c^	105.73 ± 3.79^c^	<0.01
TNF-α (pg/mL)	39.33 ± 1.06^a^	38.13 ± 0.80^b^	35.48 ± 0.92^c^	35.27 ± 0.71^c^	<0.01

^a,ab,b,c^Means in the same row with different superscripts differ (*P* < 0.05).

### 3.3 Metagenome reveals the effects of swimming exercise or a high-fat diet on gut microbiota in rats

#### 3.3.1 Quality control results of metagenomics sequencing data

Twenty-four rat cecum samples were subjected to metagenomics sequencing by Illumina sequencing platform. About 446.56 Gb of raw sequence in total was produced, and 177.47 Gb of high-quality and host-free clean reads was obtained after filtering and removing from host contamination, with an average of 7.39 Gb for each sample.

#### 3.3.2 Structural analysis of cecum flora in rats

**FIGURE 2:**
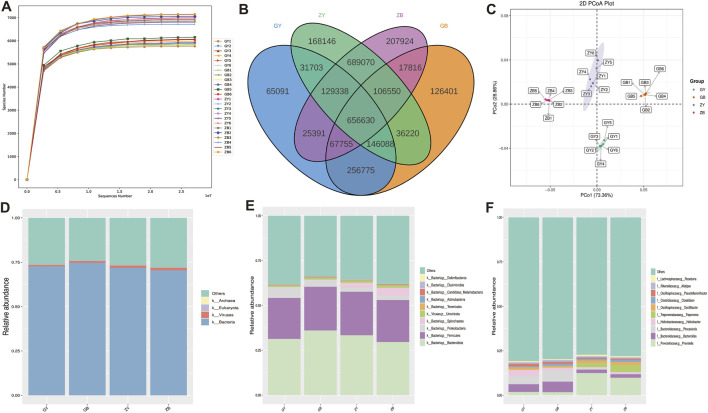
Sequencing results and species structure. **(A)** Dilution curves for all samples. **(B)** Venn diagram. **(C)** PCoA chart. **(D)** Histogram of microbial composition of the four groups at boundary level. **(E)** Bar chart of microbial composition of the four groups at the phylum level. **(F)** Histogram of microbial composition of the four groups at the genus level.

The dilution curve tends to be flat, reflecting the rationality of the amount of sequencing data, so the sequencing depth can meet the requirements for subsequent analysis (Figure 2A). The number of common features of the four groups was 656,630, and the number of unique features of the ZB group was the largest (207,924). The number of features unique to the GY group was the lowest, 65,091 (Figure 2B). As shown in Table 4, ACE, Chao1, Simpson and Shannon indexes of ZB group are significantly higher than those of other groups. The ACE and Chao1 indices of ZY group were significantly higher than those of GY and GB group. The PCoA analysis based on Bray-Curtis distance found that all samples in the same group were closer to each other, while samples in different group were farther apart (Figure 2C). At the kingdom level, the Bacteria and Viruses in the GB group were significantly higher than those in the GY group (Figure 2D). At the phylum level, Firmicutes and Bacteroidota are predominant in all samples, and the species composition of the samples in each group is basically the same (Figure 2E). At the genus level, the species composition in the same group was basically similar, but the species composition in the varied group was very different. The contents of *Bacteroides*, *Phocaeicola* and *Helicobacter* in GY group and GB group were significantly higher than those in ZY group and ZB group. The contents of *Prevotella*, *Treponema*, *Oscillibacter* and other species in GY and GB groups were significantly lower than those in ZY and ZB groups (Figure 2F).

**TABLE 4:** Cecum microbial α diversity index.

Sample	GB	GY	ZB	ZY	*P*-value
observed_species	4,582.67 ± 53.46^b^	4,432.83 ± 28.07^c^	5,199.67 ± 51.48^a^	5,165 ± 42.96^a^	<0.01
Shannon	2.13 ± 0.01^b^	2.11 ± 0.01^c^	2.21 ± 0.01^a^	2.16 ± 0.01^b^	<0.01
Simpson	0.47 ± 0.01^b^	0.48 ± 0.01^c^	0.48 ± 0.01^a^	0.46 ± 0.01^b^	0.01
Chao1	6,225.69 ± 119.32^c^	6,118.48 ± 124.73^c^	7,814.38 ± 175.73^a^	7,338.62 ± 120.71^b^	0.01
ACE	6,149.80 ± 99.49^c^	5,922.40 ± 63.41^c^	7,540.20 ± 135.21^a^	7,278.62 ± 93.87^b^	0.01

^a,b,c^Means in the same row with different superscripts differ (*P* < 0.05).

#### 3.3.3 LEfSe analysis of species differing between groups

**FIGURE 3:**
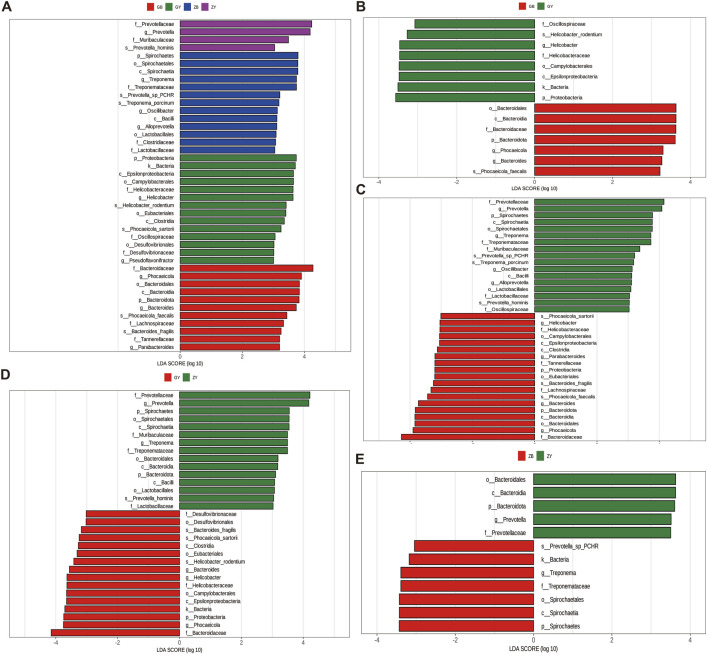
LEfSe analysis (LDA = 3, *P* < 0.05, |log_2_FC| > 1). **(A)** LDA analysis of different species in four groups. **(B)** LDA analysis of different species in GB group and GY group. **(C)** LDA analysis of different species in GB group and ZB group. **(D)** LDA analysis of different species in GY group and ZY group. **(E)** LDA analysis of different species in ZB group and ZY group.

Species with LDA Score greater than 3 were selected for LEfSe analysis among the four groups (Figure 3A). The results showed that the ZY group had relatively high content in Prevotellaceae and Muribaculaceae. The relative content of ZB group was higher in Spirochaetes and Treponemataceae. The relative content of GY group is higher in Proteobacteria, Bacteria and other species. The content of Bacteroidota, Phocaeicola and other species in GB group is relatively high. Pairwise comparisons were made between the four groups. The comparison of GB and GY groups showed that the relative content of GB group was higher in Phoceaicola, Bacteroidota and other species (Figure 3B). The comparison between GB and ZB group (Figure 3C) showed that ZB group had relatively higher contents in Prevotellaceae, Spirochaetes and other species. The comparison between ZY group and GY group showed that GY group had a relatively high content in Bacteria, Phoceaicola, Proteobacteria and other species (Figure 3D). The comparison between ZB and ZY group (Figure 3E) showed that ZY group had a relatively high content of Bacteroidia and Prevotellaceae.

#### 3.3.4 Gene function analysis

**FIGURE 4:**
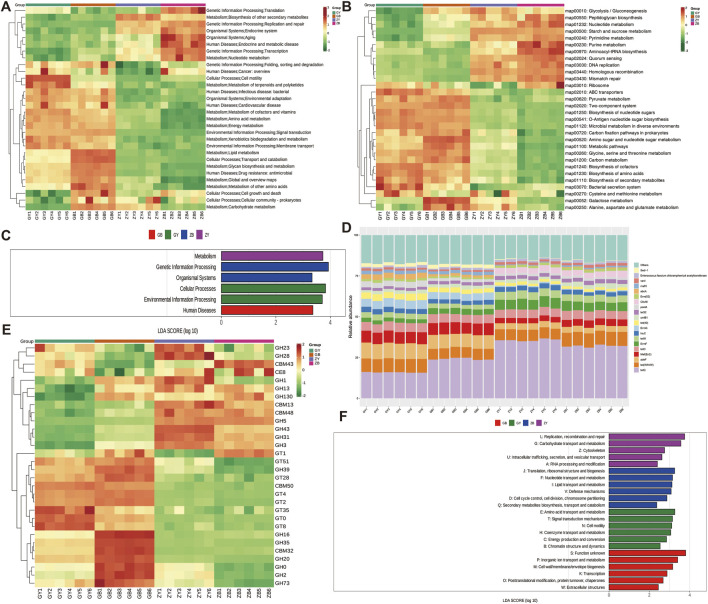
Gene function analysis **(A)** level 2 annotation of KEGG database for all samples. **(B)** All sample KEGG database level 3 level annotations. **(C)** All sample KEGG database level 1 level annotations. **(D)** CARD database annotations for all samples. **(E)** CAZy database annotations for all samples. **(F)** LEfSe analysis for the four groups based on the eggNOG database.

After annotation of KEGG PATHWAY database, the relative abundance clustering heat map (Figure 4A) was made at Level 2, and the results showed that channels such as translation, replication and repair were positively correlated with the ZB group and negatively correlated with the GB and GY groups. The relative abundance clustering heat map of all samples at Level 3 shows (Figure 4B) that Peptidoglycan biosynthesis and Nucleotide metabolism are negatively correlated with GY and GB, and positively correlated with ZY and ZB. At the same time, LDA analysis was conducted in the first category and it was found (Figure 4C) that Human Diseases were a high-abundance differential biological metabolic pathway in GB. The CARD database shows (Figure 4D) that the CfxA6 and ErmF of GY and GB groups are lower than those of the other two groups. CAZy database showed (Figure 4E) that GT2, GT4, and CBM50 were positively correlated with GY group and GB group, and negatively correlated with ZY group and ZB group. After LEfSe analysis using the eggNOG database, RNA_processing_and_modification, and lightate_transport_and_metabolism were highly enriched in the ZY group (Figure 4F).

### 3.4 The difference of cecal metabolites in rats with high fat diet or swimming exercise by widely targeted metabolomics

#### 3.4.1 All sample data quality control and structural analysis

**FIGURE 5:**
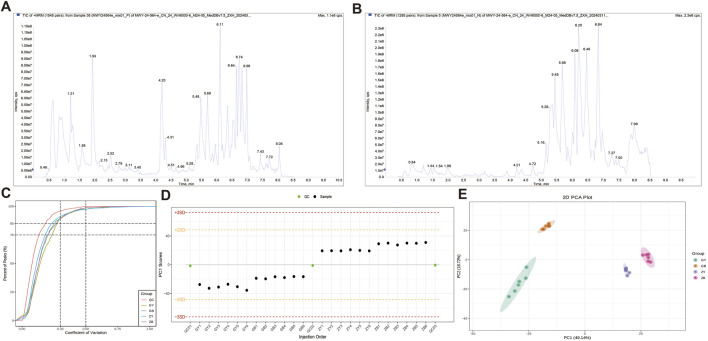
Quality control and structural analysis. **(A)** Total ion chromatogram of the detected metabolites using positive ionization mode. **(B)** Total ion chromatogram of the detected metabolites using negative ionization mode. **(C)** CV distribution diagram of each group of samples. **(D)** PC1 control diagram of the population sample. **(E)** PCA diagram of the population sample.

The metabolic analysis based on widely targeted metabolomic technology detected 1,220 metabolites. The superposition of the Total Ion Chromatogram (TIC) plots of the mass spectrometry detection of the QC samples is shown in Figure 5A,B (A: positive ion mode, B: negative ion mode). The high overlap of the curves of the TIC of metabolite detection indicates that the mass spectrometry has good signal stability when detecting the same sample at different times (Figure 5C). The proportion of substances with CV values less than 0.3 in the QC samples was higher than 85%, and the experimental data were very stable (Figure 5D). The PCA results showed a clear trend of metabolome separation between groups, with small differences between samples within each group (Figure 5E).

#### 3.4.2 Effects of swimming exercise or high-fat feeding on cecal metabolites in healthy rats

**FIGURE 6:**
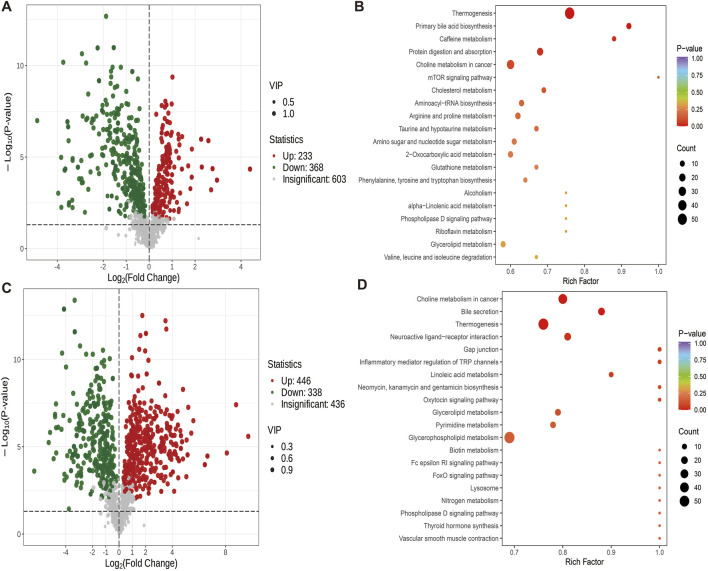
Differential metabolite volcano map (VIP>1, *P* < 0.05) and KEGG map. **(A)** Volcanic maps of differential metabolites of ZY group and ZB group. **(B)** KEGG map of different metabolites in ZY group and ZB group. **(C)** Volcanic maps of differential metabolites in groups ZB and GB. **(D)** KEGG map of different metabolites between group ZB and group GB.

The comparison between ZY and ZB group showed that 233 metabolites were up-regulated and 368 were down-regulated (Figure 6A). Compared with ZB group, ZY group showed Thermogenesis, Primary bile acid biosynthesis, Caffeine metabolism, Protein digestion and absorption, Choline metabolism in cancer and other metabolites are significantly enriched (Figure 6B). The difference metabolites of ZB group and GB group were compared. The number of up-regulated metabolites was 446, and the number of down-regulated metabolites was 338 (Figure 6C). Choline metabolism in cancer, Bile secretion, Thermogenesis, Neuroactive ligand-receptor interaction and Gap were detected in ZB group junction and other metabolites were significantly enriched (Figure 6D).

#### 3.4.3 Effects of swimming exercise on cecal metabolites in rats fed with high fat

**FIGURE 7:**
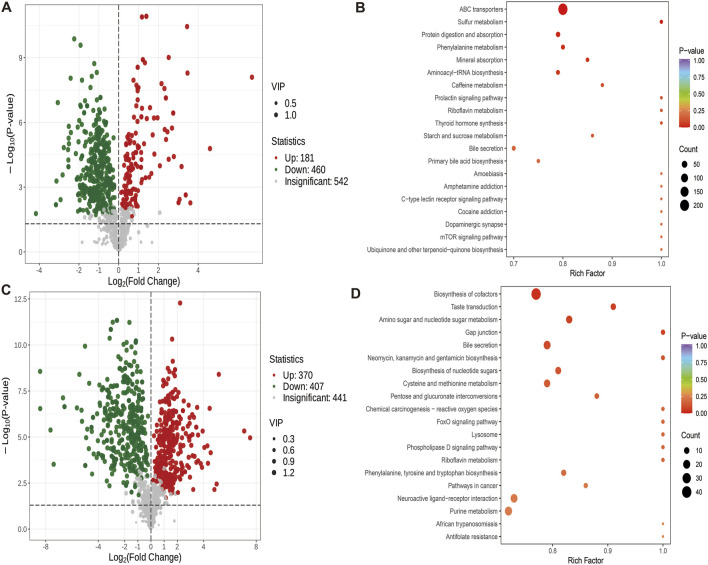
Differential metabolite volcano map (VIP>1, *P* < 0.05) and KEGG map. **(A)** Volcanic maps of differential metabolites of GY group and GB group. **(B)** KEGG map of different metabolites in GY group and GB group. **(C)** Volcanic maps of differential metabolites in groups ZY and GY. **(D)** KEGG map of different metabolites between group ZY and group GY.

A comparison between the GY and GB groups showed that 181 metabolites were up-regulated and 460 were down-regulated (Figure 7A). Compared with the GB group, the GY group exhibited significant enrichment in metabolic pathways such as ABC transporters, sulfur metabolism, phenylalanine metabolism, protein digestion and absorption, and mineral absorption (Figure 7B).

#### 3.4.4 Study on the difference of cecal metabolites between high-fat feeding and normal feeding rats under swimming exercise

The difference metabolites of ZY group and GY group were compared. The number of up-regulated metabolites was 370, and the number of down-regulated metabolites was 407 (Figure 7C). Compared with GY group, ZY group showed Biosynthesis of cofactors, Taste transduction, Amino sugar and nucleotide sugar metabolism, Gap junction and Bile Significant enrichment of secretion and other metabolites (Figure 7D).

### 3.5 Correlation analysis of differential microorganisms and metabolites

**FIGURE 8:**
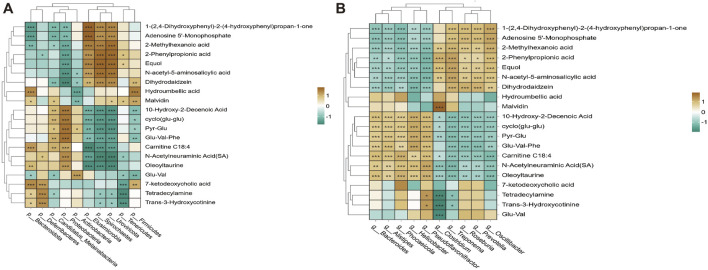
Correlation analysis of differential microorganisms and metabolites. **(A)** Analysis of the correlation between microbe and metabolites at the phylum level difference. **(B)** Analysis of the correlation between microbe and metabolites at the genus level difference.

A total of 20 differential metabolites were selected from each group and analysed for association with the top 10 microorganisms in terms of their respective abundance at the phylum and genus level, and strong correlations were found between metabolites and microorganisms (Figures 8A,B). Among them, 7-ketodeoxycholic acid and Hydroumbellic acid showed significant positive correlation with Firmicutes and Bacteroidota, but no correlation with all genus level bacteria in the top 10 abundance. *Clostridium* showed significant positive correlation with Malvidin and significant negative correlation with Trans-3-Hydroxycotinine, Tetradecylamine and Glu-Val. It was also found that Proteobacteria were significantly positively correlated with Oleoyltaurine, Glu-Val-Phe, Pyr-Glu, etc., and significantly negatively correlated with 2-Phenylpropionic acid, Equol, and Dihydrodaidzein, etc.; but interestingly, the relationship of Elusimicrobia, Spirochaeles, and Uroviricota, the three bacteria, correlated precisely with these metabolites compared to Proteobacteria.

## 4 Discussion

Analysis of the liver lesions revealed that the liver structure of the ZY group was completely normal; the liver of the ZB group was relatively normal, but mild steatosis of some hepatocytes had begun to appear; the liver of the GB group showed severe lesions, with severe steatosis and necrosis of hepatocytes as the core changes; and the liver of the GY group also showed severe lesions, but with severe vesicular degeneration of hepatocytes as the main cause, accompanied by steatosis and necrosis of part of the liver. In terms of the number of hepatocytes and the degree of steatosis, which are closely related to fat metabolism disorders, a comparison can be made, and it can be clearly shown that: the GB group (the worst) > the GY group > the ZB group; the ZY group did not have any serious lesions; the ZY group did not have any severe lesions. ZB group; ZY group had no hepatocyte steatosis. In addition, a certain degree of oedema and bruising was found on the lamina propria of the cecum of rats fed high-fat diets. Enzyme levels in rat serum, including ACP, ALP, ALT and AST, are important indicators for assessing rats’ liver health. This experiment showed that ALP, ALT, and ACP were higher in the GY and GB groups than that in the ZY and ZB groups. ALT and ALP are two enzymes found in the blood, and their elevated levels are usually associated with liver damage (Aljobaily et al., 2021). When liver cells are damaged, ALT and others are released into the bloodstream, leading to elevated levels of the corresponding enzymes in the blood (Brahmi et al., 2021). Under high-fat dietary conditions, the liver of rats may be subjected to oxidative stress and inflammatory responses, leading to an increased release of ALT and ALP, which results in elevated levels of these two enzymes in the blood. In diabetic rats, small intestinal acid phosphatase (ACP) activity may be elevated, which may be associated with diabetes-induced intestinal dysfunction (Li D. P. et al., 2023). High-fat diets are a common cause of elevated serum TC, TG, and LDL in rats. Serum TC, TG, LDL, and HDL levels are affected in rats fed a high-fat diet combined with balloon injury to the intima of the abdominal aorta in the preparation of an atherosclerosis model. In addition, a prolonged high-fat diet may lead to obesity, which further affects lipid levels. Tests showed that LDL levels decreased in high-fat-fed rats after swimming exercise, indicating that swimming exercise can consume a large amount of body heat, promote metabolism, accelerate blood circulation in the body, enhance cardiopulmonary function, and have a very good efficacy in lowering blood lipids (Müller et al., 2020). IgM is an antibody produced in the initial immune response, which has a high affinity and broad-spectrum antigen recognition ability. In rat serum, the level of IgM can reflect the immune status of the organism and the activity of certain autoimmune diseases (Mathé et al., 2024). The level of IgM in rats in the ZY and ZB groups was higher than that in the GY and GB groups, which indicates that high-fat feeding reduces the immunity of the rats. IL-1β and IL-6, as pro-inflammatory cytokines, play a key role in inflammatory reactions, activate the immune cells, promote the production of inflammatory mediators, and are involved in processes such as fever, pain and immunomodulation (Li L. et al., 2023). TNFα is a multifunctional cytokine that plays an important role in inflammation, immunomodulation, cell proliferation and apoptosis (Fang et al., 2024). In rat serum, elevated levels of IL-1β, IL-6 and TNFα are usually associated with inflammatory diseases. Our experiments showed that high-fat-fed rats reduced the probability of inflammatory diseases and promoted healthy growth after exercise.

Macrogenomic analysis of the cecum contents of 24 rats revealed that feeding high-fat diets to rats decreased the number of features in their cecums. α Diversity index showed that both groups of rats with high-fat exercise exhibited reduced richness and diversity, and PCoA analysis showed significant differences in cecum microbial structure among groups. It suggests that high-fat feeding may cause a decrease in cecum microbial species and even consequently cause metabolic disorders in rats; swimming exercise changes cecum microbial composition (Yang et al., 2023). The structural composition of their cecum microorganisms was analyzed according to different classifications, and the Viruses content in the GB group at the boundary level was significantly higher than that in the GY group, indicating that high-fat rats would reduce their viruses content after swimming exercise, which might be due to the fact that swimming exercise would change the lipid level of high-fat rats, reduce the inflammatory response, and improve the antioxidant capacity (Godoy et al., 2022). At the phylum level, Firmicutes and Bacteroidota are the main components of rat cecum microorganisms, which play a role in the rat intestine involved in digestion, metabolism and immunomodulation (Li et al., 2022). At the genus level, *Bacteroides* and *Phocaeicola* contribute to the degradation of complex heteropolysaccharides into small-chain fatty acids, and the inflammatory response of *Helicobacter* alters mammary carcinogenesis in rats (Song et al., 2023). *Prevotella* is often considered to be a bacterium associated with a healthy, plant-based diet, and to act as a “probiotic” in the human body. *Prevotella* is commonly thought to be a bacterium associated with a healthy plant-based diet and acts as a “probiotic” in the human body, and reductions in *Prevotella* have been linked to certain diseases (Li et al., 2021). *Oscillibacter* is able to metabolize cholesterol in the intestines, which helps to lower cholesterol levels, thereby reducing the risk of cardiovascular disease (Jin et al., 2022). Additionally, LEfSe analysis showed that swimming exercise increases the levels of beneficial bacteria in rats, such as Prevotellaceae bacteria that can help break down dietary fiber, produce short-chain fatty acids, maintain intestinal barrier function and modulate immune responses (Wang et al., 2022). Bacteria of the Muribaculaceae family play a key role in the response of mice to acarbose treatment. High-fat feeding reduces the content of some beneficial bacteria and causes some harmful bacteria to be produced in rats, which affects their growth (Zhao et al., 2021). In obese rats induced by high-fat diet, the relative abundance of Spirochaetes bacteria was reduced (Strnad et al., 2023), as also shown in this experiment. The Translation, Replication and repair pathway is a complex series of molecular events within the cell that are essential for maintaining normal cell function and responding to external stimuli (Alghoul et al., 2023). Peptidoglycan biosynthesis in the rat involves enzymes and proteins encoded by multiple genes, the expression and regulation of which are essential for maintenance of bacterial cell wall integrity and resistance to antibiotic attack (Rismondo et al., 2020). However, high-fat feeding decreases these metabolic pathways in rats, which may cause intestinal disturbances. CfxA6 is a gene for β-lactamases, a class of enzymes that hydrolyze β-lactam antibiotics, and the presence of the CfxA6 gene allows the bacteria to resist the action of these drugs, leading to failure of the antibiotic treatment (Lebeaux et al., 2022). The ErmF resistance gene is associated with macrolide antibiotic resistance, and it can encode an RNA methyltransferase modifies bacterial 16S rRNA, thereby making bacteria become resistant to macrolide antibiotics. The enzymes of GT2 and GT4 family in CAZy database may be related to lipid metabolism and fat deposition. The RNA processing and modification in the eggNOG database is an important step in the maturation and function of RNA molecules in cells. The Carbohydrate transport and metabolism pathway also plays an important role in rats. The demand for glucose by muscle cells increases during exercise, leading to a decrease in blood glucose levels (Gruber and Haferkamp, 2019). To maintain blood glucose levels, the liver accelerates the breakdown of glycogen and replenishes blood glucose through gluconeogenesis. In addition, exercise promotes the uptake and utilization of glucose by muscle cells to meet the energy demands of muscle contraction.

Extensively targeted metabolomic techniques were done on four groups of rats, and PCA results showed a clear trend of metabolite segregation in each group. There were a large number of differential metabolites between each group. Thermogenesis, Primary bile acid biosynthesis, and Protein digestion and absorption metabolites changed before and after swimming in normal rats. Thermogenesis metabolites are metabolites produced within an organism through metabolic processes that can promote heat production (Tang et al., 2023). Metabolites are chemical substances produced within an organism through metabolic processes that promote thermogenesis (Wu et al., 2021). In rats, the role of Primary bile acid biosynthesis involves the synthesis and secretion of bile acids that help emulsify fats during digestion and promote the absorption of fats and fat-soluble vitamins (Jiao et al., 2017). The role of protein digestion and absorption in the rat swimming assay is mainly in the maintenance of energy balance and nutritional status of rats, as well as in the absorption of drugs in the gut (Xu et al., 2024). Normal rats were more enriched in Choline metabolism in cancer, Bile secretion, Thermogenesis and other metabolites compared to high-fat-fed rats. Choline metabolism in cancer abnormally promotes cancer cell proliferation and invasion, and choline metabolites phosphorylcholine and glycerophosphoryl choline (Zhu et al., 2020). Choline and glycerophosphoryl choline are important components of cell membrane phospholipids, and their increased synthesis can support rapid division and proliferation of cancer cells (Xing et al., 2017). Bile secretion levels of cecal metabolites are altered in rats under high-fat feeding conditions. Bile acids are digestive fluids secreted by the liver into the intestines where they are involved in the process of fat digestion and absorption. Metabolites such as ABC transporters, Sulfur metabolism, and Phenylalanine metabolism were elevated after swimming in high-fat rats. Studies have shown that swimming exercise can reduce blood lipid levels and improve the lipid profile of rats, and may achieve this effect by regulating the expression and function of ABC transporters, which are involved in lipid metabolism by regulating the absorption, distribution, and excretion of lipids, thus affecting blood lipid levels and adipose tissue formation in rats. Sulfur metabolism refers to the absorption, transport, metabolism, and utilization of elemental sulfur in living organisms, of which the pyrophosphate-sulfate dehydrogenase pathway is the most important (Ward and DeNicola, 2019). Phenylalanine is an essential amino acid, which is critical for physiological processes such as protein synthesis and neurotransmitter synthesis. Studies have shown that a high-fat diet interferes with these metabolic processes, but that swimming exercise may be able to repair. Differential metabolites that were reduced in high-fat-fed rats compared to normal rats during swimming exercise were Biosynthesis of cofactors, Taste transduction, Amino sugar and nucleotide sugar metabolism (Guo et al., 2020). Biosynthesis of cofactors are key chemicals that maintain cellular redox homeostasis and drive cells to perform anabolic and catabolic reactions. They are involved in virtually all enzymatic activities that occur in living cells (Wang et al., 2018). Taste transduction is mediated by taste receptor proteins and is delivered to the nociceptors via intracellular signaling and neurotransmitter release pathways. High-fat diet-induced changes in Amino sugar and nucleotide sugar metabolism in rat cecum metabolites may be related to the composition and metabolic function of intestinal flora, which in turn affects the lipid metabolism and health status of the host (Feng et al., 2021). Amino sugars serve as a source of cellular energy and may promote cell proliferation, and nucleotide sugars may be involved in deoxyribonucleic acid replication and protein synthesis. In the analysis of differential metabolite and microbial associations, it was found that almost all metabolites were correlated with microorganisms. Secondary bile acids such as 7-Ketodeoxycholic acid (7-KCA) are converted from primary bile acids in the gut by gut microbes through the action of 7α-dehydrogenase, and they can modulate the composition and function of gut microbes and influence host metabolic and immune responses (Feng et al., 2023). Flavonoids such as Hydroumbellic acid (HUBA) have been shown to exert health benefits, such as alleviating metabolic syndrome, by modulating gut microbial composition, function and metabolism (Kayama et al., 2020). Firmicutes and Bacteroidota, on the other hand, have a profound impact on gut health and overall wellbeing through a variety of pathways, including fermentation of dietary fibre, production of short-chain fatty acids and participation in host metabolism. The strictly anaerobic nature of *Clostridium* contrasts with some of the potentially microaerobic or partially anaerobic members of Elusimicrobia and Uroviricota, which may account for their differing relevance to the same metabolites (Zhao et al., 2023). This has important implications for studies in which metabolites and microorganisms combine to influence rat health.

## 5 Conclusion

The results of this experiment revealed that the livers of high-fat-fed rats showed severe lesions, and swimming exercise had a repairing effect on the livers of rats fed in two different ways; a comparison of sections of the cecum in each group showed that the lamina propria of the cecum of the high-fat-fed rats had a certain degree of oedema and bruising. The blood levels of ALT, ALP, ACP, TC, TG, LDL, IL-6 and TNF-α were significantly higher in high-fat fed rats than in normal fed rats. The microbial richness and diversity of the cecum of high-fat-fed rats were reduced, and the microbial structures of each group were significantly different. *Bacteroides*, Phocaeicola, and *Helicobacter* microorganisms were significantly higher in the cecum of high-fat-fed rats, and Prevotella, *Treponema*, and Oscillibacter species were significantly lower. Translation, Replication and repair, Peptidoglycan biosynthesis, and Nucleotide metabolism pathways were also significantly reduced in high-fat fed rats. However, After swimming training in high-fat rats, AST, ALP, ACP, LDL, IL-1β, IL-6, and TNFα levels decreased, HDL levels increased, and Proteobacteria, Epsilonproteobacteria, and other species was relatively elevated. Metabolites such as Thermogenesis, Primary bile acid biosynthesis, and Caffeine metabolism were increased in normal rats after swimming exercise. High-fat-fed rats showed decreased levels of metabolites such as Choline metabolism in cancer, Bile secretion, and Thermogenesis compared to normal-fed rats. High-fat-fed rats had increased levels of metabolites such as ABC transporters, Sulfur metabolism, and Phenylalanine metabolism after swimming. Higher levels of metabolites such as Biosynthesis of cofactors, Taste transduction, Amino sugar and nucleotide sugar metabolism were found in normal fed rats after swimming exercise in both high fat fed rats and normal fed rats. Therefore, this experiment suggests that a high-fat diet may cause intestinal disturbances, but swimming exercise will alleviate this symptom. This test provides further theoretical support for the idea that swimming exercise will alleviate metabolic disorders caused by a high-fat diet through a multi-omics approach. It is worth noting that only rats of a single gender were selected as samples in this experiment, which can reduce variable interference, but limits the generalizability of the conclusions. Future research should include samples of both genders to improve the comprehensiveness of the study.

## Data Availability

The datasets presented in this study can be found in online repositories. The names of the repository/repositories and accession number(s) can be found below: https://www.ncbi.nlm.nih.gov/, PRJNA1136644.
